# Endoscopic mucosal resection defect inspection for predicting recurrences: International image-based survey

**DOI:** 10.1055/a-2479-8672

**Published:** 2025-01-07

**Authors:** Gijs Kemper, Ramon-Michel Schreuder, R. W.M. Schrauwen, Jochim S. Terhaar sive Droste, Peter Siersema, Erwin-Jan M. van Geenen

**Affiliations:** 1 6034 Gastroenterology and Hepatology, Radboudumc, NIJMEGEN, Netherlands; 2 3168 Gastroenterology and Hepatology, Catharina Ziekenhuis, Eindhoven, Netherlands; 3 97772 Gastroenterology and Hepatology, Bernhoven Hospital Location Uden, Uden, Netherlands; 4 10233 Gastroenterology and Hepatology, Jeroen Bosch Hospital, Den Bosch, Netherlands; 5 6993 Gastroenterology and Hepatology, Erasmus Medical Center, Rotterdam, Netherlands

**Keywords:** Endoscopy Lower GI Tract, Polyps / adenomas / ..., Endoscopic resection (polypectomy, ESD, EMRc, ...), Quality and logistical aspects, Performance and complications

## Abstract

**Background and study aims** Endoscopic mucosal resection (EMR) is a safe and minimally invasive procedure to remove colorectal non-pedunculated polyps. Recurrence rates are relatively high and differ among endoscopists. We aimed to evaluate whether endoscopists are able to predict recurrence based on thorough inspection of images of mucosal defects after an assumed complete EMR.

**Methods** We developed an online survey in which endoscopists were invited to indicate whether they expected recurrence to develop when inspecting 30 post-EMR defect images. All EMRs were considered to be complete resections by the performing endoscopist. Participating endoscopists were scored based on the number of correct answers regarding presence or absence of recurrence found at first surveillance colonoscopy.

**Results** A total of 140 endoscopists responded to the survey (response rate 25%). A total of 124 respondents with a mean age of 46.5 years evaluated the 30 images. The overall score in the cohort was 70%, indicating that respondents were able to correctly predict recurrence in three-quarters of cases with an overall level of certainty of 53.4%. When comparing results of experienced and less experienced endoscopists based on the number of endoscopic submucosal dissections and/or EMRs performed yearly, no difference (71% versus 69%, *P* = 0.23) was found.

**Conclusions** This study shows that recurrences after presumed complete EMR can reasonably well be predicted by both experienced and less experienced endoscopists when evaluating images with mucosal defects. Thorough inspection of the post-EMR defect may reduce recurrence rates by recognizing and subsequent treatment of suspect areas.

## Introduction

Endoscopic mucosal resection (EMR) is the most common treatment of large colorectal lateral spreading lesions and is considered to be safe and minimally invasive 1 . A relatively high recurrence rate (RR) of up to 20%, however, is an important drawback 2 . Considerable RRs justify a strict follow-up program. Although novel techniques focusing on the resection margin have shown to reduce RRs substantially, recurrences are still a concern 3 .

The exact mechanism of recurrences is poorly understood, yet it is likely to be multifactorial. Evidence for the assumption that unidentified residual adenoma at the resection site can lead to recurrent lesions dates from 2013 4 . It is plausible that in some cases, recurrent lesions arise from macroscopic residual neoplastic areas missed by the performing endoscopist. This may well relate to the observation that RRs among endoscopists vary greatly 4 5 . More experienced endoscopists are perhaps more successful in identifying and subsequently removing suspect residual tissue at the resection defects than non EMR-experts, resulting in lower RRs.

To assess whether recurrences are indeed associated with presence of macroscopic visible features at the resection site, we conducted a pilot study in which national and international endoscopists were invited to predict potential recurrences on images from post-EMR defects after presumed complete resection. The identification of visual features that are associated with a recurrence could highlight the importance of thorough mucosal defect inspection following EMR.

## Methods

### Image collection

We first retrospectively collected post-EMR defect images in three non-academic and one academic endoscopic center. All images comprised resection defects after EMR of polyps ≥ 20 mm in size and all procedures were considered to be macroscopically complete by the performing endoscopist. Because data were collected from electronic health records of patients, ethical approval for data collection was obtained from the regional ethical board CMO Arnhem-Nijmegen (reference number 2019–5156). The endoscopic images were anonymously exported, removing any identifying information. Images were selected by hand, based on the following criteria: (1) whole depiction of the resection defect after completion of the EMR, (2) absence of post-procedure bleeding or endoscopic instruments impairing visibility of the resection defect and (3) information about the histologic result of the post-EMR scar biopsies or resections obtained at the first surveillance colonoscopy (SC1) after 6 months. A total of 30 images were selected including six (20%) that had a recurrence at SC1 and 24 (80%) that had no recurrence at SC1.

### Survey design and participation

We built our survey with the online tool LimeSurvey GmbH. It consisted of resection defect images and additional questions related to demographics and endoscopy experience. The images were displayed randomly. Information regarding presence of a recurrence after 6 months was not provided. Potential participants were recruited in the Netherlands through the Dutch EMR study group. International endoscopists were identified by their participation in endoscopic resection guideline committees and publications on endoscopic colorectal resections. Participants were asked to examine each resection defect image for a maximum of 60 seconds and were required to indicate whether they expected recurrence to develop or not, based on resection defect features. Respondents were allowed to stop and continue the survey at any time; however, they were not able to review the image more than once so as to reflect daily clinical practice, in which sites during endoscopy are usually not reinspected.

Endoscopists were also asked to indicate whether they considered themselves to be an expert in the field of EMR and the number of EMRs and/or endoscopic submucosal dissections (ESDs) they had performed yearly in the past 3 years.

### Statistical analysis

We calculated scores for each respondent based on their correct prediction about whether the resection defect would develop recurrence at SC1 or not. A 100% score indicated that the participant was able to correctly predict presence or absence of a recurrence at SC1 on all images. Participants were also asked about their level of certainty regarding their answers. The Student’s *t* -test was used to compare mean survey scores of different groups. A one-sample *t* -test was used to reject the H _0_ , meaning that the overall score was equal to 50%, which would mean that the scores were the result of chance rather than the ability to detect potential recurrence defects based on macroscopic features. We considered *P* ≤ 0.05 significant for all analyses. Analyses were conducted using IBM SPSS Statistics version 25.

## Results

### Image selection

**Fig. 1:**
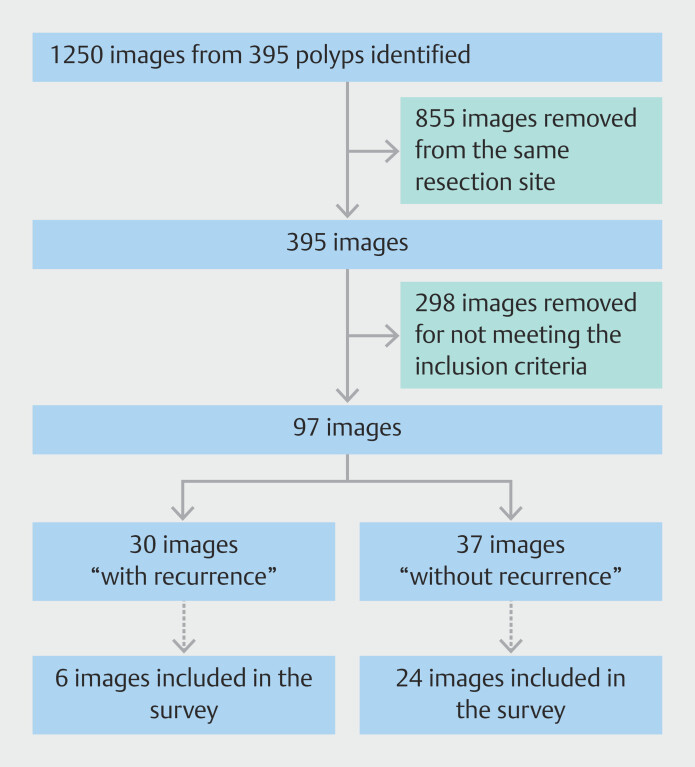
Flowchart of selection process for resection site images.

**Fig. 2:**
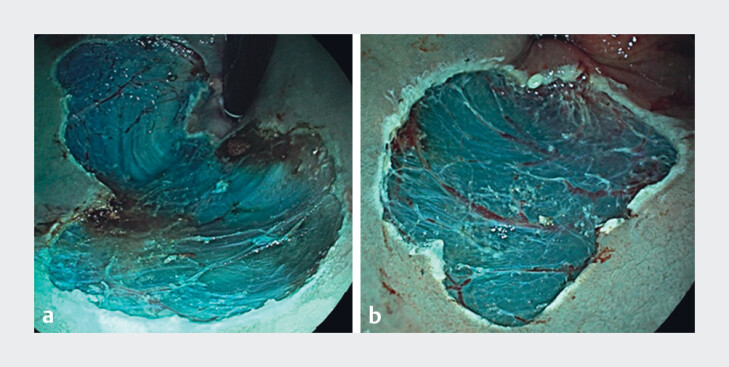
Examples of selected images. Imaging of resection site showing a recurrence and b no recurrence during follow-up.

We identified 395 polyps with 1,250 corresponding images from 2015 to 2019 in four endoscopic centers. Hand-based selection and removing duplicates (images from the same resection site) resulted in 30 images with a recurrence and 67 without a recurrence, which met our inclusion criteria. Six images with a recurrence and 24 images without a recurrence during 6-month follow-up were selected for the survey ( Fig. 1 ). Thermal ablation of the mucosal defect margins was performed in three of six (50%) with a recurrence and in 10 of 24 cases (42%) without a recurrence. Almost all resections were performed piecemeal (28 of 30, 93%). Fig. 2 shows examples of the selected images used in the survey.

### Participants

Invitations were forwarded to 558 national and international endoscopists. A maximum of two reminders were sent with an interval of 2 weeks after the first invitation. Between November 15, 2021 and December 31, 2021, 140 participants responded to the survey (response rate 25.0%) ( Table 1 ). Of these, 124 (88.6%) completed the survey, 64.5% of them being male with a mean age of 46.5 years with three-quarters working in the Netherlands (75.8%). The participants included consultant gastroenterologists (79.8%), resident gastroenterologists (15.3%), nurse endoscopists (2.4%), and one surgical endoscopist (0.8%). Most of the respondents worked at a non-academic teaching hospital (42.7%) and 37.1% at an academic hospital. Almost 50% of participants performed more than 50 EMRs or ESDs yearly and 56.5% considered themselves an EMR expert ( Table 1 ).

**Table:** **Table 1** Baseline characteristics of respondents.

**Total number of respondents (n = 140)** **Total number of respondents completed the survey (n = 124)**	
**Sex, n (%)**	
Male	80 (64.5)
Female	41 (33.1)
Non-binary	3 (2.4)
**Age in years, median (range)**	46.5 (23–68)
**Country, n (%)**	
The Netherlands	94 (75.8)
Germany	6 (4.8)
Italy	5 (4.0)
United States of America	4 (3.2)
Belgium	3 (2.4)
Portugal	2 (1.6)
Other*	9 (7.0)
Unknown	1 (0.8)
**Profession, n (%)**	
Consultant gastroenterologist	99 (79.8)
Resident gastroenterologist	19 (15.3)
Nurse endoscopist	3 (2.4)
Surgical endoscopist	1 (0.8)
Unknown	2 (1.6)
**Hospital, n (%)**	
Academic hospital	46 (37.1)
Non-academic teaching hospital	53 (42.7)
Non-academic non-teaching hospital	24 (19.4)
Unknown	1 (0.8)
**Endoscopy experience in years, mean (range)**	14.9 (2–42)
**EMR/ESD performed yearly†, n (%)**	
None	9 (7.3)
1–20	17 (13.7)
21–50	36 (29.0)
51–100	35 (28.2)
101 or more	26 (21.0)
Unknown	1 (0.8)
**EMR expert**	
Yes	70 (56.5)
No	53 (42.7)
Unknown	1 (0.8)
*France, Spain, Austria, Denmark, England, Israel, Japan, Norway, and Switzerland. †EMRs and ESDs performed yearly in the past 3 years. EMR, endoscopic mucosal resection; ESD, endoscopic submucosal dissection.	

### Scores

Participants scored an average of 69.9% for correctly detecting or excluding a recurrence ( Table 2 ). Based on the number of ESDs and EMRs performed yearly, those performing > 50 EMRs/ESDs predicted/excluded a recurrence in 71.0% and those performing ≤ 50 EMRs/ESDs ≤ 50% in 68.7% ( *P* = 0.23). Endoscopists who considered themselves an EMR expert scored 71.3% versus 67.9% in the non-expert group ( *P* = 0.08). Table 3 shows that participants scored higher when assessing resection defects resulting in recurrence versus assessing resection defects without recurrence (76.3% versus 68.3%). The overall level of certainty was 53.4% ( Table 4 ).

**Table:** **Table 2** Respondent survey score comparison.

**Total number of respondents completing the survey (n = 124)**		
	**Mean % (SD)**	***P* value **
**Overall score**	69.9 (10.9)	< 0.001*
**EMR/ESD performed yearly†, n (%)**		0.23
> 50	71.0 (11.7)	
≤ 50	68.7 (10.1)	
**EMR expert**		0.08
Yes	71.3 (9.6)	
No	67.9 (12.3)	
One-sample-test comparing the overall mean score with a score of 50. †EMRs and ESDs performed yearly in the past 3 years. EMR, endoscopic mucosal resection; ESD, endoscopic submucosal dissection; SD, standard deviation.		

**Table:** **Table 3** Respondent survey scores: Recurrence vs no recurrence.

	**Recurrence n = 6**	**No recurrence n = 24**	**Total N = 30**
Correct answer (%)	76.3	68.3	69.9
Incorrect answer (%)	23.7	31.7	30.1

**Table:** **Table 4** Respondent survey level of certainty.

**Total number of respondents completing the survey (n = 124)**		
	**Mean % (SD)**	***P* value **
**Overall level of certainty**	53.4 (17.8)	
EMR/ESD performed yearly*, n (%)		0.14
> 50	55.6 (16.4)	
≤ 50	50.9 (19.0)	
**EMR expert**		0.10
Yes	55.6 (16.6)	
No	50.2 (19.1)	
*EMRs and ESDs performed yearly in the past 3 years. EMR, endoscopic mucosal resection; ESD, endoscopic submucosal dissection; SD standard deviation.		

Endoscopists having performed > 50 EMRs/ESDs and participants who considered themselves EMR experts scored 55.6% for level of certainty compared with 50.2% and 50.9% for the non-experts and the group of endoscopists who performed ≤ 50 procedures, respectively ( *P* = 0.10 and *P* = 0.14).

## Discussion

In this survey, we evaluated whether endoscopists were able to predict adenoma recurrence based on macroscopic evaluation of images of post-EMR defects after presumed complete EMR. Endoscopists were found to correctly detect or exclude potential recurrent adenomatous tissue in 69.9%. No difference was seen between experts (performed > 50 EMRs/ESDs yearly) and non-expert endoscopists (performed ≤ 50 EMRs/ESDs yearly). These results emphasize the importance of thorough inspection of the post-EMR defect to identify potential residual adenomatous areas.

Systematically inspecting the post-EMR mucosal defect is essential for detecting evidence of an impending perforation. In addition, international guidelines state that a successful EMR is defined endoscopically by absence of neoplastic tissue after careful inspection of the mucosal defect and margin 1 6 . We hypothesized that thorough inspection of the post-EMR defect after a presumed complete EMR allows identification of areas associated with recurrence. Our findings show that careful inspection of the post-EMR defect enables endoscopists to predict recurrence correctly in more than two-thirds of cases. This demonstrates that recurrence-associated areas are visually detectable. Targeting these areas with additional coagulation or avulsion techniques may decrease recurrence rates in clinical practice 7 .

The average survey score also indicates that detectable features associated with recurrence are not always present. In these cases, recurrent lesions probably originate from undetectable microscopic neoplasia 8 . This theory is supported by recent findings in which microscopic residual adenomatous tissue was found at the defect base and margin in a macroscopic complete defect 9 . Possibly this is the result of dispersion of neoplastic cells during the EMR because it has been found that tumor manipulation during colorectal ESDs is able to exfoliate tumor cells throughout the colorectal lumen 10 . Once implanted at the resection defect, these cells can grow toward a recurrence. This could also explain why adjuvant coagulation of the resection margin reduces recurrence rates but is not able to completely prevent recurrences 5 11 12 .

We found no significant difference comparing scores of endoscopists who performed ≥ 50 EMRs/ESDs yearly with those who performed < 50 EMRs/ESDs (71.0% vs 68.7%, *P* = 0.23). Participants who considered themselves EMR experts also did not achieve significantly higher scores vs the non-experts (71.3% vs 67.9%, *P* = 0.08). These results suggest that less experienced endoscopists are also able to detect potential recurrent areas. It may, however, well be that extensive inspection of the mucosal defect attributes to lower RRs in more experienced endoscopists in clinical practice.

This study has some limitations. Most importantly, this survey used resection defect images of sometimes suboptimal quality and no video recordings were used. Moreover, assessing resection defect images without having performed the procedure makes the endoscopist unaware of important lesion and procedure characteristics. The low level of certainty in all subgroups highlights the difficulty of assessing static images.

## Conclusions

In conclusion, thorough inspection of post-EMR defect images resulted in detection of potential recurrent lesions after presumed complete resection, irrespective of endoscopist experience. These findings emphasize the value of careful inspection of the mucosal defect and its margins in clinical practice to identify potential recurrent lesions. Subsequent treatment of suspicious areas with additional therapies, in addition to coagulation of the defect margin, will likely reduce recurrence rates. Future studies are required to evaluate whether detection of potential recurrent lesion sites might benefit from other methods including artificial intelligence.
